# Glycan Structures Contain Information for the Spatial Arrangement of Glycoproteins in the Plasma Membrane

**DOI:** 10.1371/journal.pone.0075013

**Published:** 2013-09-06

**Authors:** M. Kristen Hall, Douglas A. Weidner, Jian ming Chen, Christopher J. Bernetski, Ruth A. Schwalbe

**Affiliations:** 1 Department of Biochemistry and Molecular Biology, Brody School of Medicine at East Carolina University, Greenville, North Carolina, United States of America; 2 Department of Microbiology and Immunology, Brody School of Medicine at East Carolina University, Greenville, North Carolina, United States of America; University of the Witwatersrand, South Africa

## Abstract

Glycoconjugates at the cell surface are crucial for cells to communicate with each other and the extracellular microenvironment. While it is generally accepted that glycans are vectorial biopolymers, their information content is unclear. This report provides evidence that distinct *N*-glycan structures influence the spatial arrangement of two integral membrane glycoproteins, Kv3.1 and E-cadherin, at the adherent membrane which in turn alter cellular properties. Distinct *N*-glycan structures were generated by heterologous expression of these glycoproteins in parental and glycosylation mutant Chinese hamster ovary cell lines. Unlike the *N*-linked glycans, the *O*-linked glycans of the mutant cell lines are similar to those of the parental cell line. Western and lectin blots of total membranes and GFP immunopurified samples, combined with glycosidase digestion reactions, were employed to verify the glycoproteins had predominantly complex, oligomannose, and bisecting type *N*-glycans from Pro^-^5, Lec1, and Lec10B cell lines, respectively. Based on total internal reflection fluorescence and differential interference contrast microscopy techniques, and cellular assays of live parental and glycosylation mutant CHO cells, we propose that glycoproteins with complex, oligomannose or bisecting type *N*-glycans relay information for localization of glycoproteins to various regions of the plasma membrane in both a glycan-specific and protein-specific manner, and furthermore cell-cell interactions are required for deciphering much of this information. These distinct spatial arrangements also impact cell adhesion and migration. Our findings provide direct evidence that *N*-glycan structures of glycoproteins contribute significantly to the information content of cells.

## Introduction

Glycans, like proteins and nucleic acids, are vectorial biomolecules. Unlike proteins and nucleic acids, the information content of polysaccharides encoded by the sequence of monosaccharides is unclear. *N*-Glycosylation of newly synthesized membrane proteins is in fact the most ubiquitous protein co-translational modification in the lumen of the endoplasmic reticulum [1]. An important physiochemical property conferred by membrane proteins is their lateral heterogeneity in biological membranes. Since the majority of membrane proteins are *N*-glycosylated, it is of considerable interest whether the glycans of *N*-glycosylated membrane proteins contain information about their association, clustering, and distribution in the cell membrane. Up to date, the information of the *N*-glycans of glycoproteins has been shown to affect important physiochemical properties such as conformation, stability, protease resistance, charge, and water binding capacity [2]. Congenital disorders of glycosylation (CDG) in humans [3] and mutant glycosylation mice [4], [5], [6] emphasize the importance of the *N*-glycosylation process in the context of a multicellular organism. Clearly, establishing the cellular information content of *N*-glycans would tremendously assist in understanding their role in the development and maintenance of an organism.

E-Cadherin and Kv3.1 are transmembrane glycoproteins which have critical biological roles. E-Cadherin is the primary adhesion molecule involved in calcium-dependent cell-cell interactions [7]. It has four utilized *N*-glycosylation sites and removal of these sites generates very unstable protein [8]. Further, abrogation of the two sites closest to the *N*-terminus of E-cadherin altered calcium-dependent cell-cell adhesion, while vacancy of the site closest to the C-terminus had an immense influence on protein stability. Kv3.1 is a voltage-gated K^+^ channel which has two absolutely conserved *N*-glycosylation sites [9]. Vacancy of these *N*-glycosylation sites produced functional Kv3.1 channels at the cell surface [10]. However, ion conductance and neuronal-derived cell migration were altered [11]. As such, it is of considerable interest to determine the information content of the glycans associated with the E-cadherin and Kv3.1 glycoproteins.

In an initial and simplified approach to explore the role of *N*-linked glycan structures on membrane glycoprotein distribution, and subsequent effects on cell-cell contacts, we examined the spatial arrangement in the adherent plasma membrane of two integral membrane glycoproteins, Kv3.1 and E-cadherin, and subsequently explored their roles in cell adhesion and migration. *N*-Glycosylated proteins contain three types of *N*-glycans: oligomannose, hybrid, and complex [12]. Further the core mannose of hybrid and complex *N*-glycans can be modified by the addition of a bisecting *N*-acetylglucosmine (GlcNAc) which is referred to as bisecting type *N*-glycans. We generated different forms of both glycoproteins by heterologous expression of each of the proteins in parental (Pro^-^5) and glycosylation mutant (Lec1 and LEC10B) Chinese hamster ovary (CHO) cell lines. The Pro^-^5 cell line produces the majority of *N*-glycosylated proteins with complex *N*-glycans while the major glycoproteins produced by the Lec1 and LEC10B CHO cell lines are those with oligomannose *N*-glycans and bisecting type *N*-glycans, respectively [13], [14]. The *O*-glycans of the mutant cell lines are similar to those of the Pro^-^5 cell line. We observed that the distribution of Kv3.1 and E-cadherin in the adherent plasma membrane of two or more cells was altered by changing the structure of *N*-glycans associated with either of the glycoproteins in both a glycan-dependent and protein-dependent manner. For single cells, these differences revealed less dependence on the protein and on the *N*-glycan attached to the glycoprotein while the glycoconjugates were more influential. Increases in the concentration of the Kv3.1 and E-cadherin glycoproteins at the cell-cell contact correlated with enhanced cellular migratory rates and cell adhesiveness, respectively. Taken together, the interpretation of the information encoded by the type of *N*-glycan was drastically different between the various forms of the Kv3.1 and E-cadherin glycoproteins when cell-cell interactions existed while the differences were quite subtle for these glycoproteins in the absence of cell-cell interactions.

## Materials and Methods

### Recombinant vectors

To construct Kv3.1-pEGFP-N3 and N220Q/N229Q-pEGFP-N3 recombinant vectors, Polymerase Chain Reaction was employed to remove stop site and add BamHI sites on 5′ and 3′ ends. Kv3.1-pCDNA 3.1 and N220Q/N229Q- pCDNA 3.1 recombinant vectors were used as templates [15]. E-cadherin-GFP expression vector (GenBank Accession # L08599) was purchased (Addgene, Cambridge, MA, USA).

### Cell culture and transfections

Parental Pro^-^5 and glycosylation mutant Pro^-^Lec1 (Lec1) CHO cells were obtained from American Type Culture Collection (Manassas, VA, USA). Glycosylation mutant cell line Pro^-^LEC10B (LEC10B) were gifted by Dr. Pamela Stanley, Albert Einstein College of Medicine, New York [13]. Cells were maintained in MEM Alpha Media (Hyclone, Logan, UT, USA) supplemented with 10% fetal bovine serum, 50 U/mL penicillin and 50 µg/mL streptomycin (Gemini BioProducts, West Sacramento, CA, USA) at 37°C under 5% CO_2_. For the production of stable cell lines, CHO cells of 60–70% confluency were transfected with neomycin selectable expression plasmids encoding wild type Kv3.1, N220Q/N229Q Kv3.1, and E-cadherin as previously described [11].

### Total membrane isolation

CHO cells (≈1.35 X 10^8^) were homogenized in lysis buffer (10 mM Tris, pH 7.4; 250 mM sucrose, 5 mM EDTA; protease inhibitor cocktail set III (Calbiochem, San Diego, CA, USA) 1∶500), and centrifuged at 2,000× g for 10 min. Supernatant was centrifuged at 100,000× g for 1 h. Pellet was resuspended in lysis buffer and protein concentration was determined by Lowry assay. Samples were stored at −80°C.

### Glycosidase digestions of total membranes

Glycosidase digestions of total CHO membranes were conducted. Total membranes (5 g/L) were treated with 20 U/µL PNGase F, 50 U/µL Endo H and 0.83 U/µL neuraminidase in appropriate buffers (New England Biolabs, Ipswich, MA, USA). Reactions were left overnight at 37°C and stopped by adding reducing SDS-PAGE sample buffer.

### Immunoprecipitation of GFP fusion proteins

Transfected CHO cells (3–7×10^7^ cells) were sonicated in lysis buffer (50 mM sodium phosphate, pH 7.4, 0.3 M potassium chloride, 0.5% triton X-100; protease inhibitor 1∶500), and centrifuged at 1,000× g for 15 min. Gel slurry of anti-GFP conjugated to agarose (Medical & Biological Laboratories, Nagoya, Japan) was added to low speed spin supernatant and rotated at room temperature for 1 h. Resin was washed twice with 50 mM sodium phosphate, pH 7.4, 0.3 M potassium chloride, and PBS by spinning at 660× g for 5 minutes. Reducing SDS sample buffer was added to equal amounts of gel slurry containing GFP fusion proteins. Samples were incubated overnight at room temperature, and immediately used for lectin blotting or stored at −20°C.

### Western blot analysis

Kv3.1 and E-cadherin total membrane samples were electrophoresed for 1.7 h and 3.5 h at 20 mAmps on 10% and 12% SDS gels, respectively. Electrophoresed proteins were transferred to PVDF membranes (Millipore, Billercia, MA, USA) for 3 h at 100 V or 4 h at 250 mAmps, respectively. For GFP immunopurifed samples, proteins were electrophoresed for 1.7 h at 20 mAmps on 10% gels and proteins were transferred for 3 h at 250 mAmps. Incubations and development of blots, as well as monoclonal mouse anti-Kv3.1 (Neuromab, Davis, CA, USA), were as described [16]. Rabbit pan Cadherin antibody (Novus Biologicals, Littleton, CO, USA) was utilized to detect E-cadherin. Western Blots were performed at least three times for each transfected cell line, except for the LEC10B N220Q/N229Q, which was tested two times. All results were consistent.

### Lectin blotting

Total membranes (25 µg) or immunopurified GFP-Kv3.1 and GFP-cadherin samples were electrophoresed for 1.7 h at 20 mAmps on 10% SDS gels and separated proteins were transferred to membranes for 3 h at 250 mA. Blots were incubated in blocking buffer (PBS, 5% non-fat dry milk (Bio-Rad) with 0.05% Tween 20) followed by incubation with biotinylated L-PHA, E-PHA, or GNL (Vector Labs, Burlingame, CA, USA). Blots were washed four times with PBS plus 0.05% Tween 20. Membranes were incubated with strepavidin conjugated to alkaline phosphatase for 1 h at room temperature. Blots were washed four times with PBS plus 0.05% Tween 20, and twice with PBS, and developed with ImmunO alkaline phosphatase substrate (MP Biomedicals, Irvine, CA, USA). Membranes containing electrophoresed total membrane proteins were stained with coomassie blue. Lectin blots of total membranes were tested a minimum of two times while lectin blots of GFP immunopurified samples were tested a minimum of three times. In all cases, the results were reproducible.

### TIRF microscopy

Stable transfected cells were isolated using a FACS Vantage (Becton Dickinson, Franklin Lakes, NJ, USA) cell sorter with laser excitation at 488 nm and green fluorescence emission at 515–545 nm and seeded onto 35 mm poly-L-lysine coated glass bottom dishes (MatTek, Ashland, MA, USA) for about 26 h. Live cells were excited with an argon laser beam of wavelength 488 nm entering the side illumination port of an Olympus IX-71 microscope (Olympus, Center Valley, PA, USA) through a Apo 60X 1.45 objective and images captured with an ORCA R2 deep cooled mono CCD camera. Detection settings were kept constant. Exposure time of 1000 ms was utilized for data analysis. The shutters, filters, camera, and data acquisition were controlled by CellˆTIRF Control 1.1 and Metamorph for Olympus Basic software. Image J software was utilized to measure the fluorescence intensity signal for the relative amount of protein in the membrane patch, including that at the cell-cell border, and it was also used to measure the number and size of particles in the various regions of the membrane patch. The number of particles (size 5–10,000 pixels) in the total adherent patch, the exterior border of the membrane patch, and the interior plasma membrane, excluding the cell-cell interface, was determined. For the percentage of particles in the interior or exterior plasma membrane, the number of particles in each of the respective areas of the plasma membrane was divided by the particles in the total adherent patch.

### Wound healing assays

Cell migration experiments were conducted as described [11]. Cells were seeded in equal concentrations. Upon cell confluency, media was removed and wounds were made in the monolayer using a beveled 200 µl pipet tip. Cells were rinsed twice with media. Images were obtained at 0 and 16 h on an Olympus IX 50 microscope using a 10X objective. The percent of closure increase due to the *N*-glycan attached to Kv3.1 was determined by taking the difference in wound closure between glycosylated (W_gly_) and unglycosylated (W_ungly_), and dividing the difference by the closure of unglycosylated (W_ungly_) using the following equation: 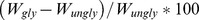.

### Dissociation assays

Dissociation assays [17] were modified. Cells were seeded in equal amounts on 35 mm dishes. Upon cell confluency, cells were washed twice. Cells were removed by one complete rotation with a cell scraper. Cells were dissociated by pipetting seven times. Images (25 fields/dish) were obtained on an Olympus IX 50 microscope using a 10X objective. Particles, cell aggregates with more than five cells, were counted and particle size measured. Data are presented as the difference between the area of Cadherin-CHO particles (A_Cad_) and nontransfected CHO particles (A_non_), and then dividing the difference by the area of nontransfected CHO particles, as indicated in the equation: 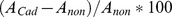.

### Data analysis

Image J software was used for mean fluorescence intensity, particle size and number. Adobe Photoshop was utilized for wound size measurements. Origin 7.5 was used for graphics and statistics. Data is presented as the mean ± S.E. where *n* denotes the number of cell areas measured. The unpaired student's *t*-test was utilized when comparing populations and a value of *p*<0.05 was considered significant, unless otherwise indicated. All microscopy experiments were performed on at least three different days.

## Results

### Type of glycans attached to Kv3.1 and E-cadherin in various CHO cells

**Figure 1:**
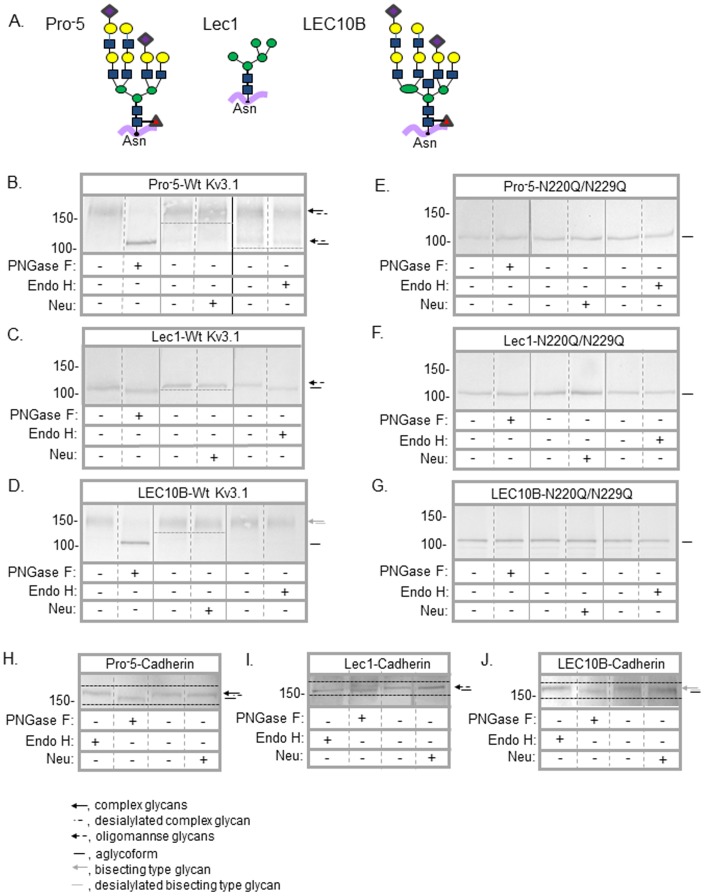
Western blots of Kv3.1 and E-cadherin glycoproteins expressed in various CHO cell lines. Diagram depicting the predominant *N*-linked glycans present in each CHO cell line studied (A). Symbolic nomenclature is presented as outlined by the Consortium for Functional Glycomics Nomenclature Committee as follows: ▪, *N*-acetylglucosamine; •green circle, mannose; •yellow circle, galactose; ♦, sialic acid; ▴, fucose. Western blots of wild type (Wt) and N220Q/N229Q Kv3.1 proteins, and E-cadherin glycoprotein digested (+) and undigested (−) with PNGase F, Endo H, and neuraminidase (neu) when heterologously expressed in Pro^-^5 (B,E,H), Lec1 (C,F,I), and LEC10B (D,G,J) cells, as indicated. Dashed lines below and/or above the immunobands on the various panels were employed to emphasize the small electrophoretic shifts. Solid black vertical lines denote different blot. The numbers adjacent to the Western blots represent the Kaleidoscope markers (in kDa).

Two and four *N*-glycans are attached to wild type Kv3.1 [18] and E-cadherin [8] glycoproteins, respectively. Removal of both *N*-glycosylation sites of the Kv3.1 protein generates functional unglycosylated N220Q/N229Q Kv3.1 protein [11], [18] while abolishment of all four sites of E-cadherin produces virtually no protein at the cell surface [8]. As such, the aglycoform of E-cadherin generated by site directed mutagenesis could not be evaluated. To examine whether *N*-glycan occupancy and structure impacts spatial arrangements of Kv3.1 and E-cadherin in the plasma membrane, wild type Kv3.1, N220Q/N229Q Kv3.1, and E-cadherin proteins tagged with EGFP were heterologously expressed in a parental CHO cell line (Pro^-^5) and two *N*-glycosylation mutant CHO cell lines (Lec1 and LEC10B). Lec1 and LEC10B cells generate glycoproteins with oligomannose *N*-glycans and bisecting type *N*-glycans, respectively, while Pro^-^5 cells produce predominantly complex *N*-glycans (Figure 1A) [13], [14]. We isolated total membranes from the various CHO cells expressing wild type Kv3.1, N220Q/N229Q Kv3.1, and E-cadherin proteins. These membranes were treated without (−) and with (+) PNGase F (removes complex, hybrid and oligomannose *N*-glycans), neuraminidase (neu, cleaves sialyl residues from non-reducing termini of carbohydrate chains) or Endo H (removes oligomannose), and then were analyzed by Western blots (Figure 1). The immunoband of wild type Kv3.1 isolated from Pro^-^5 cells (Figure 1B; ≈153 kDa) migrated much slower than that from Lec1 cells (Figure 1C; ≈111 kDa) and slower than that from LEC10B cells (Figure 1D; ≈142 kDa). In some cases, a small percent of the Kv3.1 glycoprotein from Pro^-^5 cells migrated to a similar position as that expressed in Lec1 cells, and this immunoband migrated slightly faster upon treatment with Endo H (Figure 1B). This result indicates that some of the glycoprotein escaped processing in the *cis*-Golgi. In all cases, N220Q/N229Q (Figure 1E–G; ≈106 kDa) isolated from the various cell lines migrated faster than the wild type Kv3.1, and the migration was virtually identical in all cell lines. Further the electrophoretic migration of N220Q/N229Q was similar to those of wild type Kv3.1 from Pro^-^5, Lec1 and LEC10B cells treated with PNGase F, as well as wild type Kv3.1 from Lec1 cells treated with Endo H. When wild type Kv3.1 isolated from either Pro^-^5 or LEC10B cells was treated with neuraminidase, small increases in electrophoretic migration could be observed. In contrast, this small shift was undetected for wild type Kv3.1 expressed in Lec1 cells, as well as N220Q/N220Q from all three cell lines.

We also analyzed total membranes from Pro^-^5 (Figure 1H), Lec1 (Figure 1I) or LEC10B (Figure 1J) CHO cells expressing E-cadherin on Western blots. We found that E-cadherin expressed in all three cell groups treated with PNGase F caused an increase in electrophoretic mobility while only E-cadherin expressed in Lec1 cells migrated faster when treated with Endo H. Electrophoretic mobility shifts generated by neuraminidase were quite subtle for E-cadherin expressed in either Pro^-^5 or LEC10B cell lines. Further, the electrophoretic migration appeared slightly slower for E-cadherin isolated from Pro^-^5 cells (≈168 kDa) than that from LEC10B cells (≈165 kDa). E-cadherin from Lec1 cells (≈161 kDa) was the smallest. Therefore, these results demonstrated that the greater part of the Kv3.1 and E-cadherin glycoproteins from Pro^-^5 and LEC10B cells had complex *N*-glycans while oligomannose *N*-glycans composed these proteins from Lec1 cells. Further the major Kv3.1 glycoprotein expressed in the Pro^-^5 cells was different relative to that from LEC10B cells since the electrophoretic migrations were dissimilar, suggesting that the *N*-glycans of Kv3.1 expressed in LEC10B were of bisecting type. Importantly, these results also revealed that Kv3.1 and E-cadherin proteins tagged with GFP were folded correctly since the *N*-glycans were processed in the Pro^-^5 and LEC10B cell lines.

**Figure 2:**
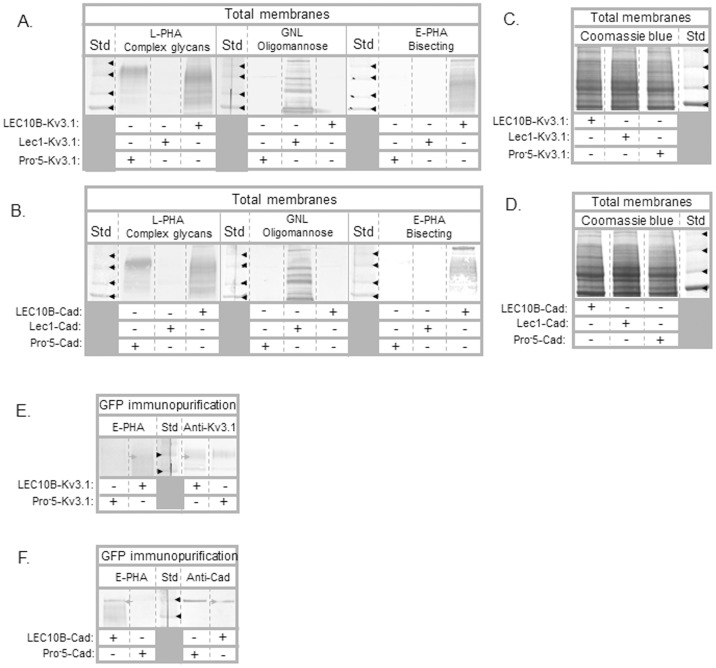
Lectin blots of total membranes and immunopurified Kv3.1 and E-cadherin proteins from transfected CHO cell lines. Total membranes (∼25 µg) from Pro^-^5, Lec1, and LEC10B cells transfected with wild type Kv3.1 (A) and E-cadherin (B) were probed with L-PHA (∼5 µg/mL), E-PHA (5–10 µg/mL), and GNL (∼10 µg/mL). Similar amounts of electrophoresed proteins from total membranes were also stained with Coomassie blue (C,D). Black arrowheads denote the 75, 100, 150 and 250 kDa markers. Lectin blots of immunopurified GFP tagged Kv3.1 and E-cadherin from transfected Pro^-^5 and LEC10B cells (E,F). Glycoproteins were probed with E-PHA (5–20 µg/mL). Western blots were run in parallel to denote position and relative amount of GFP-Kv3.1 and E-cadherin protein. Grey arrowheads point to GFP tagged Kv3.1 (E) and E-cadherin (F) proteins expressed in LEC10B cells while black arrowheads represent the 100 and 150 kDa markers.

To further define the major type of *N*-glycans attached to the various Kv3.1 and E-cadherin glycoproteins expressed in Pro^-^5, Lec1 and LEC10B cell lines, we conducted lectin blotting of total membranes and GFP immunopurified samples. We identified complex, oligomannose, and bisecting type *N*-glycans of glycoproteins by utilization of the following lectins: *Phaseolus vulgaris* Leucoagglutinin (L-PHA), *Galanthus Nivalis* Lectin (GNL), and *Phaseolus vulgaris* Erythroagglutinin (E-PHA), respectively [13]. As expected, lectin blots of total membranes revealed that by far the majority of the glycoproteins expressed in either the Kv3.1 (Figure 2A) or E-cadherin (Figure 2B) transfected Pro-5, Lec1 and LEC10B cell lines consisted of complex, oligomannose and bisecting type *N*-glycans, respectively. Further the majority of glycoproteins expressed in both transfected LEC10B cells also interacted with L-PHA while they did not interact with GNL, indicating that the majority of glycoproteins with bisecting type *N*-glycans were complex *N*-glycans. In both cases, relative amounts of total membrane proteins loaded were similar, as shown by coomassie blue staining of PVDF membranes (Figure 2C and 2D).

Lectin blots of immunopurified GFP tagged Kv3.1 (Figure 2E, lane 2) and E-cadherin (Figure 2F, lane 1) showed that E-PHA interacted with glycoproteins from Kv3.1 and E-cadherin transfected LEC10B cells, respectively. Alternatively, E-PHA interactions were unobserved from Kv3.1 (Figure 2E, lane 1) and E-cadherin (Figure 2F, lane 2) transfected Pro^-^5 cells. Adjacent Western blots revealed that lectin staining was observed at a similar position as the immunoband of the Kv3.1 glycoprotein expressed in LEC10B cells (Figure 2E, lane 4), and that the top lectin stained band was at a similar position as the E-cadherin immunoband from E-cadherin transfected LEC10B cells (Figure 2F, lane 5). Lectin blots, along with Western blots and glycosidase digestion reactions, revealed that the major form of either of Kv3.1 or E-cadherin glycoproteins expressed in Pro^-^5, Lec1 and LEC10B cell lines consist of complex, oligomannose and bisecting type *N*-glycans, respectively. These results are in agreement with previous studies of these CHO cell lines [13], [14]. As such, we will refer to the predominant form of wild type Kv3.1 and E-cadherin glycoproteins as composed of complex, oligomannose and bisecting type *N*-glycans from Pro^-^5, Lec1 and LEC10B cells, respectively, and furthermore the N220Q/N229Q Kv3.1 protein as unglycosylated Kv3.1 protein throughout the main text and figures.

### Localization of the Kv3.1 glycoprotein to the cell-cell border

**Figure 3:**
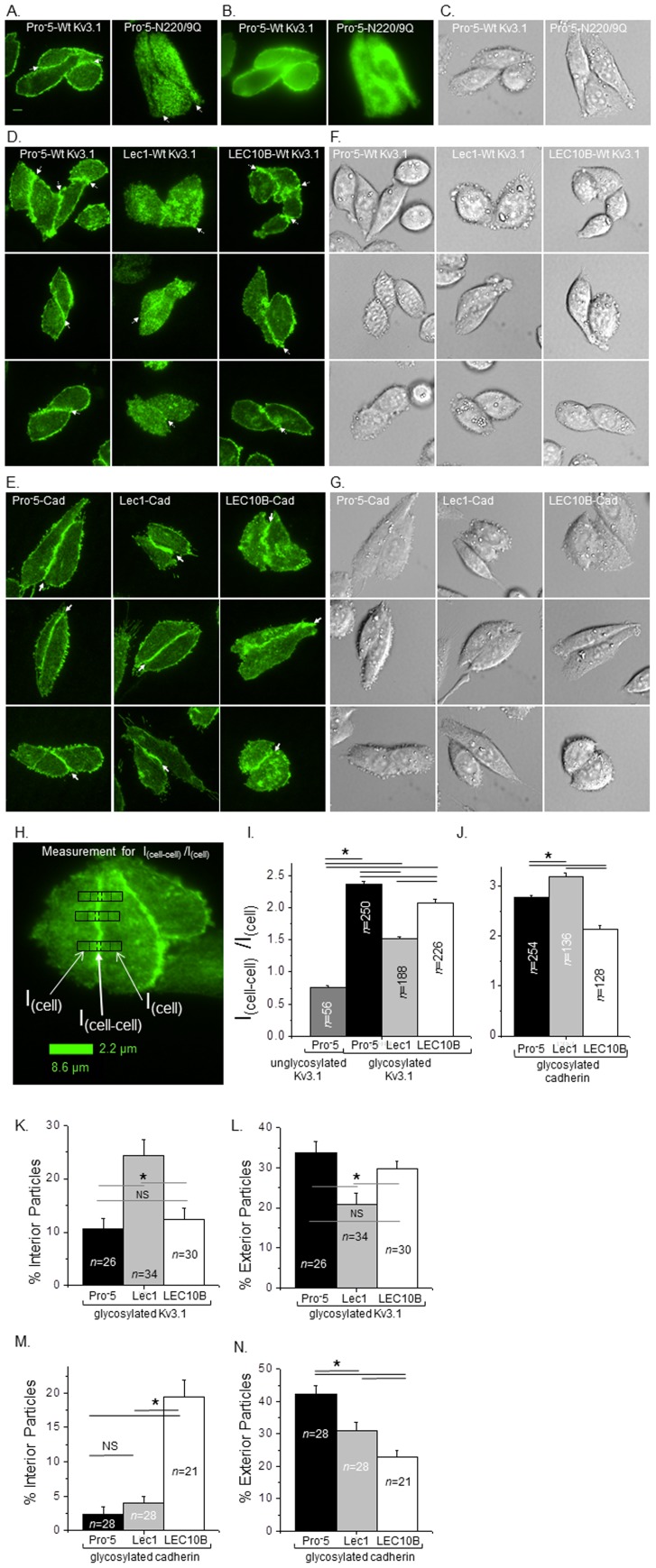
Variations in the glycosylation pathway impact the localization of Kv3.1 and E-cadherin at the cell-cell border. Microscopy images were acquired in TIRF (A), wide-field (B), and DIC (C) modes for EGFP tagged wild type (left panels) and N220Q/N229Q (right panels) Kv3.1 proteins expressed in Pro^-^5 cells. TIRF (D,E) and DIC (F,G) images of the wild type Kv3.1 (D,F) and E-cadherin (E,G) proteins expressed in Pro^-^5 (left panels), Lec1 (middle panels), and LEC10B (right panels) cells. Representative scale bar (5 µM) was identical for all images. White arrows point to cell-cell interface. The enlarged TIRF image illustrates the measurements for determining the amount of Kv3.1 and E-cadherin at the cell-cell interface (H). Fluorescence intensity measurements were determined at the cell-cell interface (I_cell-cell_), and away from the cell-cell interface (I_cell_) of the cell membrane patch. White arrows point to designated regions in the black rectangle with four dividing lines. The scale bar represents the size of the black rectangles. The bar graph reports the I_cell-cell_/I_cell_ of N220Q/N229Q (unglycosylated) and wild type (glycosylated) Kv3.1 proteins (I), as well as E-cadherin (J), expressed in the various CHO cell lines. At the 0.000001 level, the differences of the population means are significantly different by one-way ANOVA with Bonferroni adjustment (*). The percent of particles located in the interior (K,M), excluding the particles at the cell-cell interface, and exterior (L,N) regions of the membrane patch was calculated for two interacting cells. At the 0.02 and 0.03 level, the differences of the population means are significantly different by student *t*-test (*) for the Kv3.1 and E-cadherin proteins, respectively. NS denotes samples are not significantly different.

We employed total internal reflection fluorescence (TIRF) microscopy to acquire high contrast images of live Pro^-^5 cells expressing glycosylated (left panel) and unglycosylated (right panel) Kv3.1 tagged with EGFP at the plasma membrane (Figure 3A). Alternatively, images acquired from the same channel after modifying the laser beam to attain wide-field fluorescence excitation showed more diffuse and dimmer signals (Figure 3B). Of note, the endoplasmic reticulum and nucleus were clearly visible in the wide-field images, and quite lacking in the TIRF images. Fluorescence intensity signals from TIRF images versus wide-field images verified that the signals from TIRF images were of higher intensity (mean fluorescence intensity values of TIRF images to mean fluorescence intensity values of wide-field images were 1.42±0.02, *n* = 41 and 1.39±0.04, *n* = 18 for Pro^-^5 cells expressing glycosylated and unglycosylated Kv3.1, respectively). Further these results support that images could be obtained in TIRF mode to examine greater details of the spatial location of Kv3.1 in or near the adherent plasma membrane. Differential interference contrast (DIC) images were obtained in the same plane to identify the position of the cells in TIRF images (Figure 3C). Fluorescence intensity signals were quite strong at the cell-cell interface, as well as the exterior regions of the membrane patch, for Pro^-^5 cells expressing glycosylated Kv3.1, while the signals were distributed throughout the entire patch with perhaps less signal at the cell-cell border for those expressing unglycosylated Kv3.1. These results verified expression of glycosylated and unglycosylated Kv3.1 in the plasma membrane [11], [18], [19], and furthermore that the *N*-glycans of Kv3.1 contributes to its localization at the cell-cell border.

### Glycan structures of Kv3.1 and E-cadherin impacts localization to cell-cell border

Changes in the glycosylation pathway of Lec1 and LEC10B cells led to the production of different forms of the Kv3.1 and E-cadherin glycoproteins than those expressed in the Pro^-^5 cells. We compared TIRF microscopy images of Kv3.1 (Figure 3D) and E-cadherin (Figure 3E) glycoprotein from Pro^-^5 (left panels), Lec1 (middle panels), and LEC10B (right panels) cells. In all cases, DIC images of Kv3.1 (Figure 3F) and E-cadherin (Figure 3G), as well as wide-field images (not shown), were acquired to correlate fluorescence signals at specific positions within the cell patch. Fluorescence intensity signals were quite strong at the cell-cell interface, and outer regions of adhered membrane patches, for Kv3.1 expressed in LEC10B cells, similar to that expressed in Pro^-^5 cells. On the contrary, when Kv3.1 was associated with oligomannose *N*-glycans, the fluorescence intensity signal was detected at the cell-cell border but to a much lesser degree than the other forms of Kv3.1, and furthermore the distribution of the signal appeared to be greater in the interior plasma membrane region of each cell of the membrane patch.

For each form of the E-cadherin glycoprotein, fluorescence intensity signals were more concentrated at the cell-cell border than other regions of the adherent membrane patch. Additionally, the E-cadherin glycoprotein expressed in Pro-5 and Lec1 cells had very little fluorescence signal in the interior plasma membrane region of each cell of the membrane patch while the signal from E-cadherin transfected LEC10B cells was found in substantial amounts in this region. The exterior regions of the cell membrane patch had quite high fluorescence intensity signal for Pro^-^5 and Lec1 cells expressing E-cadherin while the signal in the E-cadherin transfected LEC10B cells were considerably lower. Of note, if one of the two interacting cells did not express E-cadherin then it was not observed at the cell-cell border (Figure 3E, top panel of middle row). This observation supports that the predominant interaction of E-cadherin is homotypic, instead of heterotypic [7].

To further characterize the distribution of the glycoproteins in the cell membrane, we determined the ratio of the fluorescence intensity signal at the cell-cell interface (I_cell-cell_) of the membrane patch to that away from this interface (I_cell_) for TIRF images (Figure 3H). This measurement revealed that Kv3.1 with sialylated complex *N*-glycans was more strongly localized to the cell-cell border than that with bisecting type *N*-glycans and much stronger than that with oligomannose *N*-glycans, and furthermore that the aglycoform was more prevalent at other regions of the adherent membrane patch (Figure 3I). Of note, this ratio of unglycosylated Kv3.1 expressed in Pro^-^5 cells (I_cell-cell_/I_cell_ was 0.75±0.03, *n* = 56) was similar to those in the Lec1 and LEC10B cells expressing the unglycosylated Kv3.1 (I_cell-cell_/I_cell_ was 0.72±0.03, *n* = 68; and 0.79±0.03, *n* = 64 for Lec1 and LEC10B cells, respectively). As such, this data suggests that the distribution of unglycosylated Kv3.1 in adhered membrane patches was insensitive to glycoconjugates at the cell surface. On the contrary, the amount of E-cadherin localized to the cell-cell border was higher in Lec1 cells than Pro^-^5 cells, and lowest in LEC10B cells (Figure 3J). Therefore, these results support that distinct *N*-glycan structures attached to Kv3.1 and E-cadherin glycoproteins, as well as the individual protein structure, were responsible for the differential distribution of the glycoproteins at the cell-cell border in the adhered membrane patch of groups of cells.

To elaborate on the information captured by TIRF images, we determined values of the number of particles in total adherent membrane patch, in interior plasma membrane of the patch with exclusion of the cell-cell interface, and in exterior border of the membrane patch. The percent of interior membrane particles for the Kv3.1 glycoprotein with either sialylated complex or bisecting type *N*-glycans were at least 2-fold lower than that with oligomannose *N*-glycans (Figure 3K) which correlated with the higher percent of exterior membrane particles for the earlier two forms of the glycoprotein (Figure 3L). Mean area of the particles in the total membrane patch for Kv3.1 expressed in Lec1 cells (80±13 pixels, *n* = 14) was smaller than those expressed in Pro^-^5 (143±19 pixels, *n* = 12) and LEC10B (136±19 pixels, *n* = 15) while the number of particles in the total membrane patch for Kv3.1 expressed in Lec1 cells (55±7 particles, *n* = 14) was slightly larger than those expressed in Pro^-^5 (48±6 particles, *n* = 12) and LEC10B (49±6 particles, *n* = 15). Further the particle size and number were about 2-fold larger in exterior regions compared to interior regions of the latter two transfected cell lines while in the former cell line, these values were quite similar throughout. Mean intensity values of the particles from total membrane patches for Pro^-^5, Lec1, and LEC10B were not significantly different for the various forms of Kv3.1.

The E-cadherin glycoprotein expressed in the various cell lines also revealed differences in the percent of particles in the various regions of the membrane patch. E-cadherin with bisecting type *N*-glycans had at least 5-fold more E-cadherin particles localized to the interior region of the membrane patch than E-cadherin with complex or oligomannose *N*-glycans (Figure 3M). Further the amount of E-cadherin with complex *N*-glycan structures was significantly greater at the exterior region of the membrane patch than that with oligomannose *N*-glycans, and about 2-fold greater than that with bisecting type *N*-glycans (Figure 3N). The area and mean intensity of E-cadherin particles in total membrane patches, and designated interior and exterior regions of patches were not significantly different in the various CHO cell lines. Taken together, these results revealed that *N*-glycan structures, along with protein structure, guide the spatial arrangement of various forms of the Kv3.1 and E-cadherin glycoproteins in adherent membrane patches of groups of cells.

### Distribution of glycoproteins in single cells

**Figure 4:**
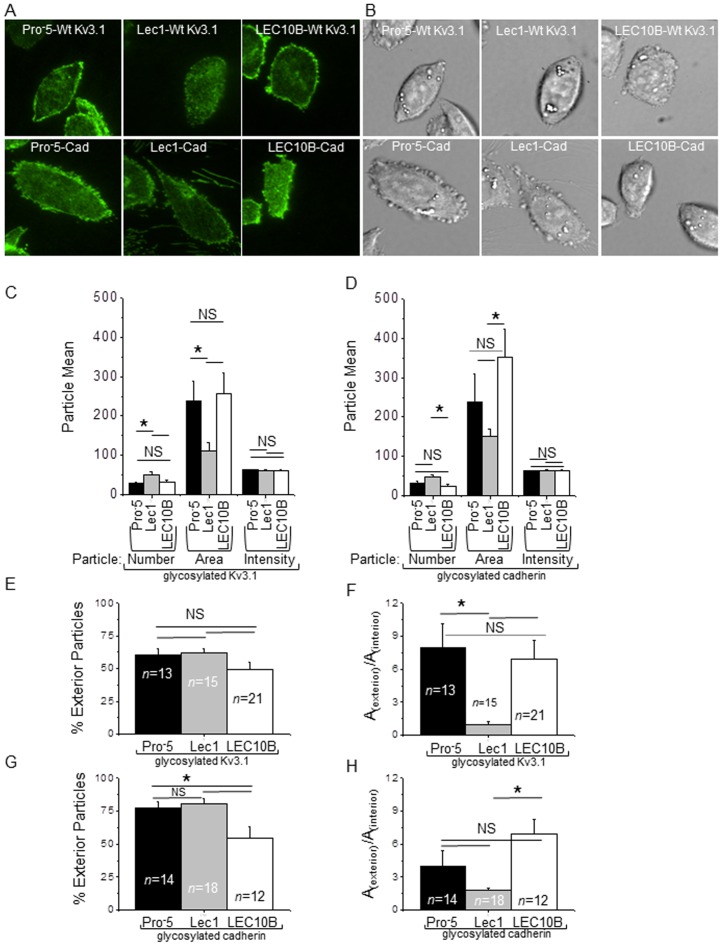
Distribution of Kv3.1 and E-cadherin at the cell surface of single cells. TIRF (A) and DIC (B) microscopy images of Pro^-^5 (left panels), Lec1 (middle panels) and LEC10B (right panels) cells expressing either Kv3.1 (upper panels) or E-cadherin (lower panels) are shown. Representative graphs of the mean particle numbers, areas (pixels), and intensities (AU) for Kv3.1 (C) and E-cadherin (D) are shown. The percent of particles located in the exterior region of the cell for Kv3.1 (E) and E-cadherin (G) was determined. The particle area in the exterior of the cell relative to the particle area in the interior of the cell for Kv3.1 (F) and E-cadherin (H) was calculated.

Since *N*-glycan structures of both Kv3.1 and E-cadherin were shown to have an impact on their spatial arrangement in a cell which interacted with other cells, we ascertained whether this occurred in single cells. Representative TIRF (Figure 4A) and DIC (Figure 4B) microscopy images of single Pro^-^5 (left panels), Lec1 (middle panels) and LEC10B (right panels) cells expressing either Kv3.1 (upper panels) or E-cadherin (lower panels) are shown. In all cases, the fluorescence signal was more concentrated on exterior regions of the cell membrane patches than on interior regions. The general trend was that Lec1 cells expressing either Kv3.1 (Figure 4C) or E-cadherin (Figure 4D) had a greater number of particles and the size of the particles was smaller than those from transfected Pro^-^5 and LEC10B cells. In all cases, the mean intensity of the fluorescence was quite similar. When comparing particles in interior regions of the membrane patches to those at the edge or exterior regions, a similar pattern was detected for cells transfected with either Kv3.1 (Figure 4E,F) or E-cadherin (Figure 4G,H). However, there were a greater percentage of particles in the exterior region for E-cadherin transfected Pro^-^5 and Lec1 cells than those from Kv3.1 transfected Pro^-^5 and Lec1 cells. Further the size of the particle in exterior regions relative to the interior was significantly greater for the E-cadherin transfected Lec1 cells than those from Kv3.1 transfected Lec1 cells. Of note, differences were not observed between the Lec10B cells transfected with either Kv3.1 or E-cadherin. Taken together, glycoconjugates at the cell surface appeared to dominate the distribution of the glycoproteins in the adhered plasma membrane of single cells. However, subtle changes due to a given membrane glycoprotein could be detected in the transfected Pro^-^5 and Lec1 cells.

### Cell migratory rates are influenced by glycans of Kv3.1

**Figure 5:**
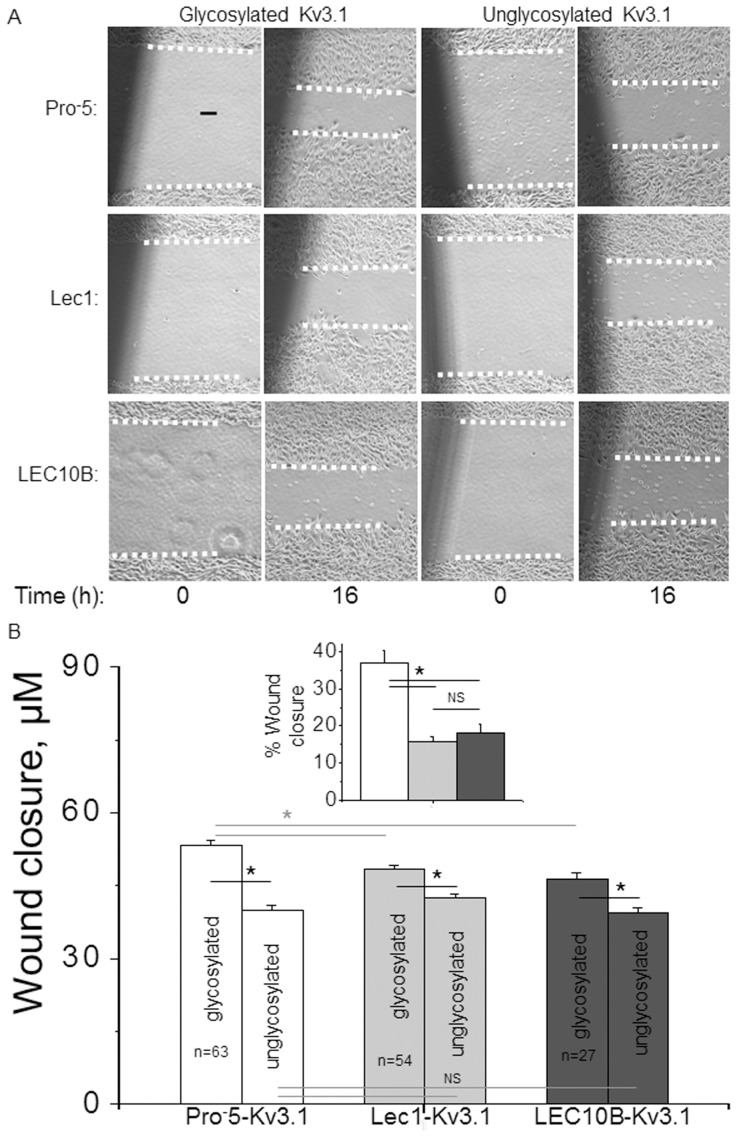
Rate of CHO cell migration is enhanced by Kv3.1. Cell wounds were generated for glycosylated and unglycosylated Kv3.1, and then images were captured at 0 and 16 h time points for Pro^-^5 (top row), Lec1 (middle row), and LEC10B (bottom row) (A). Rate of cell wound closure was determined for glycosylated and unglycosylated Kv3.1 expressed in the various CHO cell lines (B). Region of wound measured is indicated by the two dotted white lines per image. The percent of wound closure is the increase in wound closure due to the *N*-glycan attached to Kv3.1 (B, inset). Asterisks indicate significant differences in mean values at a probability of *P*<0.03.

Recently, it was shown that neuroblastoma cells heterologously expressing glycosylated Kv3.1 migrate faster than those expressing unglycosylated Kv3.1 [11]. We therefore ascertained whether different *N*-glycan structures of distinct Kv3.1 glycoproteins could alter cell migration since their distribution in the plasma membrane was different. We performed cell wound healing assays for glycosylated and unglycosylated Kv3.1 expressed in Pro^-^5, Lec1 and LEC10B cells (Figure 5). Similar cell wound sizes (about 83 µm) were monitored at 0 h and 16 h for glycosylated and unglycosylated Kv3.1 expressed in Pro^-^5 (top row), Lec1 (middle row), and LEC10B (bottom row) cells (Figure 5A). Cell wound closures were greater for glycosylated Kv3.1 than its unglycosylated counterpart in each of the distinct cell lines (Figure 5B). Further the migratory rates for glycosylated Kv3.1 from both Lec1 and LEC10B cells were slower than that in Pro^-^5 cells while they were not different for the aglycoform among the various cell lines. To illustrate how the structure of *N*-glycans of Kv3.1 altered cell migration, we determined the percent of closure increase due to the *N*-glycan attached to Kv3.1 by taking the difference in wound closure between glycosylated and unglycosylated Kv3.1, and then dividing the difference by the closure of unglycosylated Kv3.1 (Figure 5B, Inset). These results revealed that sialylated complex *N*-glycans of Kv3.1 contributed to a greater degree to cell wound closure than either oligomannose *N*-glycans or *N*-glycans with bisecting GlcNAc residues. Therefore, this study indicated that *N*-glycan structures associated with Kv3.1 can impact cell migratory rates.

### *N*-Glycans of E-cadherin alter cell-cell adhesion

**Figure 6:**
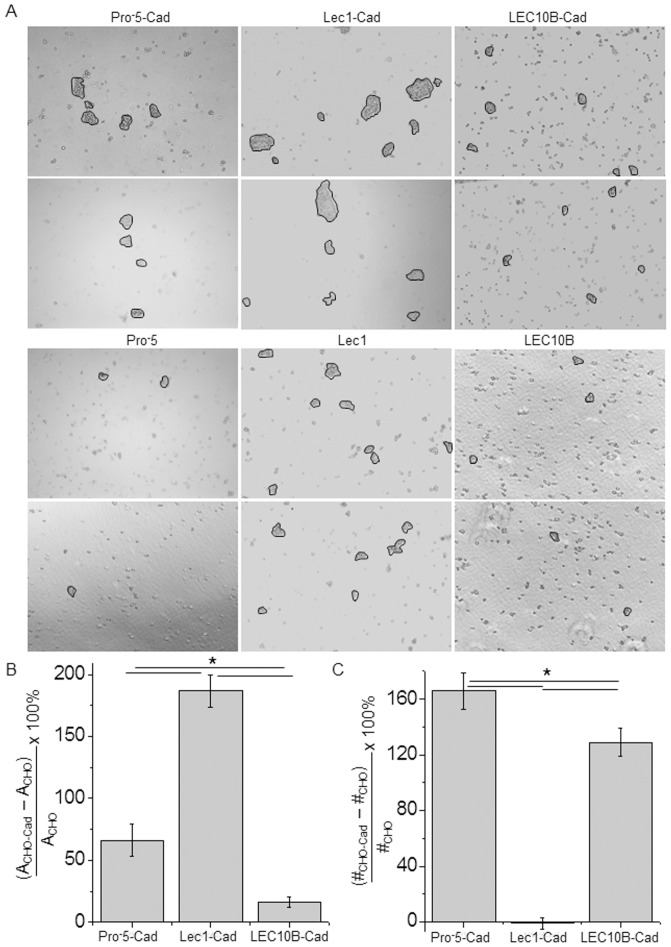
Glycan structures of E-cadherin alter cell dissociation. Microscopy images were acquired for Pro^-^5 (left panels), Lec1 (middle panels), and LEC10B (right panels) cells transfected with E-cadherin (upper panels) and nontransfected (bottom panels) (A). Particles of interest are encircled. Percents of particle area (B) and particle number (C) represent increases in the mean values of the particles between the E-cadherin transfected CHO cells and the corresponding nontransfected CHO cells, *n* = 125 images. Asterisks indicate significant differences in mean values at a probability of *P*<0.03.

TIRF microscopy measurements showed that E-cadherin with oligomannose *N*-glycans had more E-cadherin at the cell-cell interface than that with complex *N*-glycans and much more than that with bisecting type *N*-glycans. As such, we anticipated that Lec1 cells transfected with E-cadherin would have stronger cell-cell interactions than E-cadherin transfected Pro^-^5 cells and much stronger than the E-cadherin transfected LEC10B cells. Representative images from cell dissociation assays are shown for Pro^-^5 (left panels), Lec1 (middle panels) and LEC10B (right panels) cells expressing E-cadherin (upper two panels) and those similar cells not transfected (lower two panels) (Figure 6A). These images revealed that E-cadherin transfected Lec1 cells (11069±517 pixels, *n* = 1086) had larger particles (>5 cells/aggregate) than E-cadherin transfected Pro^-^5 cells (5306±416 pixels, *n* = 529) and much larger than E-cadherin transfected LEC10B cells (2463±88 pixels, *n* = 717). A similar size pattern was observed for their nontransfected counterparts (Lec1, 3851±162 pixels, *n* = 1029; Pro^-^5, 3195±351 pixels, n = 195; LEC10B, 2123±90 pixels, *n* = 320). However, the percent of increase in particle area for E-cadherin transfected Lec1 cells was about 3-fold and 12-fold greater than transfected Pro^-^5 and LEC10B cells, respectively (Figure 6B). Further the percent of increase of particle area was about 4-fold greater for transfected Pro^-^5 cells than transfected LEC10B cells. Next, we determined the percent of increase in the number of particles to be higher for E-cadherin transfected Pro^-^5 cells than transfected LEC10B cells while the particle number was unchanged for transfected Lec1 cells (Figure 6C). Of note, the number of particles for nontransfected cells was highest for Lec1 (8.2±0.3 particle number/image, *n* = 125), and lowest for Pro^-^5 (1.6±0.1 particle number/image, *n* = 125) while LEC10B (2.5±0.2 particle number/image, *n* = 125) had intermediate levels. A similar trend was observed for CHO cells transfected with E-cadherin (Lec1, 8.1±0.4 particle number/image, *n* = 125; LEC10B, 5.7±0.2 particle number/image, *n* = 125; Pro^-^5, 4.3±0.2 particle number/image, *n* = 125). Overall, these results showed that the type of *N*-glycans at the cell surface could influence the size and number of cell aggregates upon similar disruption procedures of the various CHO cell monolayers. Further transfection of the various CHO cell lines with E-cadherin had an even greater impact on the size and number of the particles. In this regard, the E-cadherin glycoprotein with oligomannose *N*-glycans had stronger cell-cell interactions than that with complex *N*-glycans and much stronger than that with bisecting GlcNAc *N*-glycans.

## Discussion

**Figure 7:**
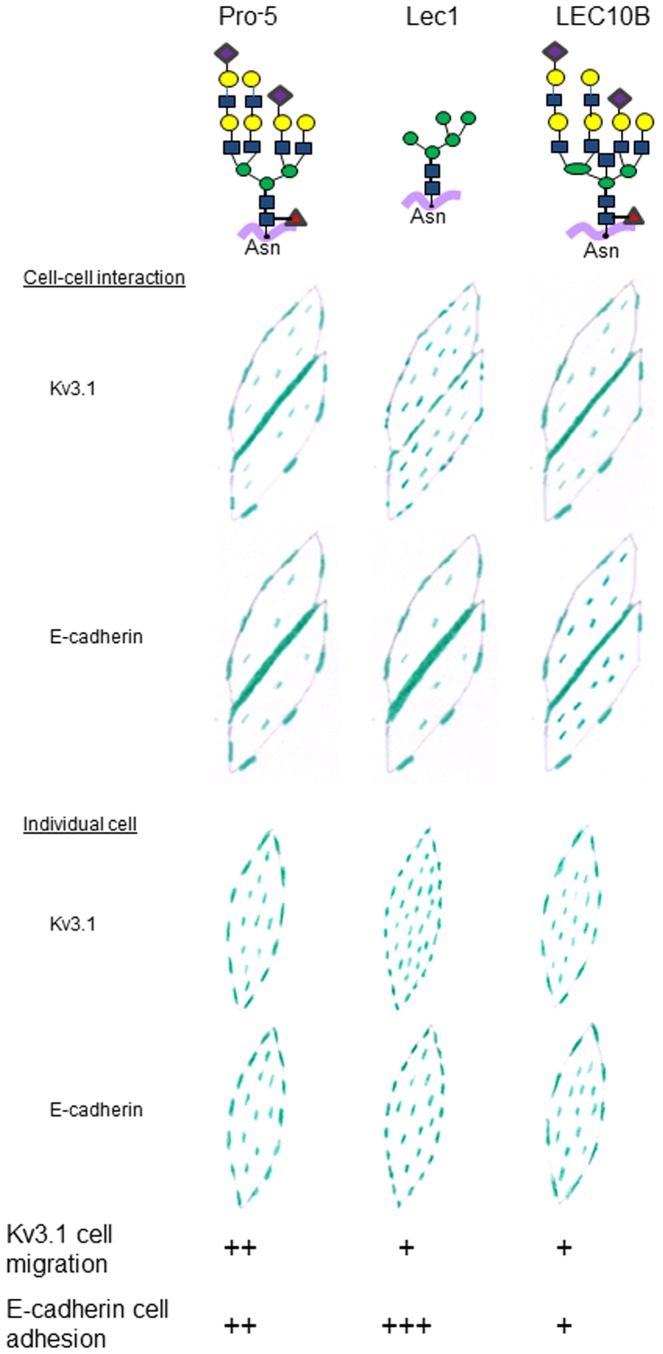
Model depicting the information encoded by glycan structures of Kv3.1 and E-cadherin. Top drawings show predominant glycans present in each CHO cell line studied [14]. In the cell drawings, green dashes denote the relative amounts of GFP tagged Kv3.1 or E-cadherin particles in the adhered membrane of interacting (Figure 3) and non-interacting (Figure 4) cells. The relative effects of the glycoproteins on cell migration (Figure 5) and cell adhesion (Figure 6) are shown with (x) signs.

It has long been appreciated that membrane proteins contain information about their association, clustering, and distribution in the plasma membrane of cells. However, the importance of *N*-glycan structures of membrane glycoproteins on their lateral heterogeneity in biological membranes is not well known [20]. Herein we report that changes in *N*-glycan structures of two distinct glycoproteins had a major impact on their distribution in the adherent plasma membrane when two or more cells interacted with each other (Figure 7). Further these changes in spatial arrangement due to *N*-glycan structure were much less pronounced for single cells. This remarkable difference illustrates that the information contained by *N*-glycans of glycoproteins is crucial for cells to communicate with each other. Heterologous expression of either Kv3.1 or E-cadherin in Pro^-^5, Lec1 and LEC10B cell lines was employed to produce major forms of each glycoprotein with complex, oligomannose and bisecting type *N*-glycans. Without exception, all forms of each of the glycoproteins conferred different distribution patterns. These patterns were also dependent on the protein since the glycoprotein with oligomannose or complex *N*-glycans had the highest levels of E-cadherin and Kv3.1 at the cell-cell border, respectively. On the other hand, the lowest levels of E-cadherin and Kv3.1 at the cell-cell border were observed for the glycoprotein with bisecting type *N*-glycans or oligomannose *N*-glycans, respectively. In both cases, these lowest levels correlated with the highest levels of the glycoproteins in the interior regions of the membrane patch. These results support that the *N*-glycans are informational biomolecules that relay information for the spatial arrangement of glycoproteins in the plasma membrane, and furthermore cell-cell interactions are required for deciphering much of this information.

We showed that the major forms of both glycoproteins were consistent to the type of *N*-glycan expressed in each CHO cell line, such as complex, oligomannose and bisecting type. Kv3.1 has two sites which are processed to complex *N*-glycans [9] with a minimal amount evading glycan processing in the *cis*-Golgi of the Pro^-^5 cell line. E-cadherin has four utilized sites [8], and our results indicated that the occupied sites had complex *N*-glycans when expressed in Pro^-^5 cells. Further expression of E-cadherin with oligomannose *N*-glycans was undetected in this cell line. Taken together, the fine-tuning of cell-cell interactions remains to be defined with regards to *N*-glycan multiplicity and microheterogeneity of *N*-glycans at the various sites.

Control of the spatial arrangement of glycosylated transmembrane proteins at the cell surface is a critical aspect of cellular function. As the number of occupied *N*-glycosylation sites, combined with branched *N*-glycans, was increased for a given membrane glycoprotein, then more of the protein was concentrated in the plasma membrane [21]. Enrichment of the glycoproteins in this type of microdomain occurs since crosslinking of the glycoproteins to the galectins delays endocytosis. Binding studies of galectins with various CHO cell lines showed that as *N*-glycan branching is decreased then the galectin-glycan interactions are reduced [22]. We observed that the area of the particles was smaller for the single transfected Lec1 cells than the transfected Pro^-^5 and LEC10B cells, as well as Kv3.1 with oligomannose *N*-glycans in groups of cells. Next, differences were detected at cell adhesion junctions. Particle sizes of Kv3.1 and E-cadherin at the cell-cell border were larger relative to other regions in the adherent membrane, and the level of each of the glycoproteins at this border was dependent on the glycan structure. Therefore, these results indicate that the content of two distinct microdomains in the plasma membrane depends on the *N*-glycan structure and primary sequence of the glycoprotein. As such, it is critical to consider the role of the *N*-glycans in modulating movement of glycoproteins in the plasma membrane.

*N*-Glycosylation processing of E-cadherin from oligomannose *N*-glycans to complex *N*-glycans is a critical factor in determining the strength of cell-cell interactions. E-cadherin with increased levels of oligomannose *N*-glycans or reduced occupancy of the *N*-glycosylation sites promotes the establishment of stable adhesion junctions while increased occupancy and complex *N*-glycans significantly weakened these junctions [23]. Our results showed that cells expressing E-cadherin with oligomannose *N*-glycans had more E-cadherin at the cell-cell border than that with complex *N*-glycans, and furthermore the higher concentration of E-cadherin at the cell-cell border enhanced cell-cell adhesion. These findings explain the earlier study, and directly reveal that distinct *N*-glycan structures of E-cadherin provide information for tuning cell-cell interactions. It has also been proposed that cell-cell interactions were stronger when Mgat3 expression was increased due to enhanced cell surface expression of the E-cadherin and larger cell aggregates were observed by the classical cell association assay [24]. This proposal is in contradiction to the amount of E-cadherin at the contact site between cells for Pro^-^5 cells relative to the LEC10B cells. However, our spatial arrangement of E-cadherin with bisecting type *N*-glycans could result in larger cell aggregates since there was more E-cadherin away from the cell-cell border. Thus, our results show a direct correlation between the level of E-cadherin at the cell-cell border and strength of the cell-cell interaction. As such, *N*-glycan structures of E-cadherin contain information about the strength of the calcium-dependent homotypic interactions between cells.

Predominant glycan structures have been implicated as critical determinants for defining the stage and path of tumor progression. Knockdown of Mgat5 in mice [25], as well as Mgat1 knockdown in cells [26], results in a decrease in tumor incidence, growth and metastasis. More recently, it was proposed that certain forms of the E-cadherin glycoprotein can increase metastasis and progression of malignancy [27], [28]. In this regard, E-cadherin expressed in cells that increase the number and degree of β1,6-GlcNAc branched *N*-glycans enhance tumor progression and metastasis while increases in E-cadherin with oligomannose *N*-glycans minimize these processes. Our study evolves this mechanism by directly demonstrating that E-cadherin with oligomannose *N*-glycans localizes to the cell-cell border to a greater extent than that with complex *N*-glycans. Further this higher expression correlated with an increase in cell-cell adhesion. Taken together, cancer cells producing glycoproteins with complex *N*-glycans, instead of oligomannose *N*-glycans, are more likely to detach from the originating tumor and invade neighboring tissues since less E-cadherin is directed to the contact site between cells.

Modifications of the *N*-glycan structures of Kv3.1 have critical effects on cell migration. Previous studies from our lab showed that neuroblastoma cells heterologously expressing Kv3.1 with two *N*-glycans migrated faster than those with one *N*-glycan, and much faster than unglycosylated Kv3.1 [11]. In the present study, it was verified that cells expressing unglycosylated Kv3.1 migrated slower than those expressing glycosylated Kv3.1, and furthermore cells expressing Kv3.1 with complex *N*-glycans migrated faster than cells expressing Kv3.1 with either oligomannose *N*-glycans or *N*-glycans with a bisecting GlcNAc residue. Since enhanced cell migration is a feature of malignant transformation and an increased ratio of complex *N*-glycan structures to oligomannose *N*-glycans accompanies tumor progression [26], it may be that increased expression of the Kv3.1 channel in cancerous cells [29] impacts cellular migratory rates.

We conclude that *N*-glycan structures contain information that guides the spatial arrangement of a voltage-gated potassium channel and a major adhesion molecule in the plasma membrane. The information encoded by the different *N*-glycan structures for both transmembrane glycoproteins were intensified when cells were interacting, opposed to individual cells. The *N*-glycosylation process has a significant impact in mammalians while this process can be obliterated in single cells without deleterious consequences. As such, our results provide insight into how aberrant changes in the *N*-glycosylation process prove detrimental in mammalian development, growth, and disease progression.
